# Measuring health-related quality of life in tuberculosis: a systemic review - Response

**DOI:** 10.1186/1477-7525-8-7

**Published:** 2010-01-19

**Authors:** Stephen E Weis, Jotam G Pasipanodya

**Affiliations:** 1 University of North Texas Health Sciences Center at Fort Worth, 3500 Camp Bowie Blvd, Fort Worth, Texas 76107, USA; 2 Division of Infectious Diseases, UT Southwestern Medical Center, Dallas, Texas, 5323 Harry Hines Blvd, Dallas, Texas 75390-9113, USA

## 

We read with interest the recently published paper by Na Guo et al [1]. The authors identified published studies [2-4] where health-related quality of life instruments were administered to persons with tuberculosis. They then analyzed the health-related quality of life structure, validation, and health-related quality of life outcomes. The authors' conclude that the consensus of the published health-related quality of life literature is that tuberculosis significantly negatively impacts patients' quality of life. They further found that even after being microbiologically 'cured' of TB that health-related quality of life remained significantly worse than the general population. We agree with the authors' major conclusions that tuberculosis significantly negatively impacts patients' quality of life. We also concur that the qualitative evidence they present suggests these impacts persists after microbiological 'cure' of tuberculosis disease.

We however did not completely understand their statement in results, "A validated tuberculosis-specific quality of life instrument was not located". Their methods used to determine if an instrument was "validated" were not stated. As the authors are aware, the ideal methods for validation of health-related quality of life questionnaires are controversial. We recently used the St George's Respiratory Questionnaire to ascertain health-related quality of life in treated TB patients. We used a widely validated generic health-related quality of life instrument and biological measures for construct validity in TB patients. Further most items in the St George's instruments have sound theoretical and practical relevance and have been validated for similar respiratory diseases [2,5]. Additionally, our review of these [2-4] and other literature suggests that the instruments used were adequately translated and majority of them were validated. As the authors correctly pointed out, as instruments become more disease or organ-specific they invariably become less sensitive to the broad goals of measuring health outcomes in communities. Additionally, there are inherent biases in subjective judgments that should be considered when people are asked opinions about their health. However, as long as the instruments are sensitive enough to detect differences that are clinically important, they remain valuable tools for evaluating clinical outcomes and measure disease burden, especially in resources-constrained communities.

There has been an increase in international standardization of health-related quality of life measurements. Banks of items that measure health constructs rather than health-related quality of life questionnaires have been developed [5]. Using these items the ceiling and floor effects common with most health measuring instruments can be minimized. We concur with Ian McDowell that in the future these banked items can then be used for designed specific purposes with ease of comparability since they will have been psychometrically evaluated. Ideally these instruments can then be validated using evidence-based guidelines so discussion can focus on the results of health-related quality of life studies not the test itself.

Measurement of health is contentious because of the complexity and abstract nature of health itself [5]. Despite these controversies data on health outcomes, especially among populations affected by illnesses that cause low mortality rates, are crucial for shaping health care policy [5]. Tuberculosis in low tuberculosis-burden areas is an example of such an illness. Mortality is rare but as the authors analysis demonstrates poor health persists despite microbiological cure of tuberculosis disease. As a result tuberculosis has a greater effect on population health than is apparent from incidence and mortality data. We appreciate the authors' review of this topic and find from their analysis convincing support for expanding LTBI treatment guidelines. Currently only treatment of latent tuberculosis infection (LTBI) can prevent pulmonary impairment after tuberculosis (PIAT).

## Competing interests

Both authors have no completing interests in the subject discussed.

## Authors' contributions

Conceived and designed the study; JGP, SEW.

Collecting data; JGP, SEW.

Analyzed data; JGP, SEW.

Write manuscripts; JGP, SEW.

Both authors read and approved the final manuscript.
